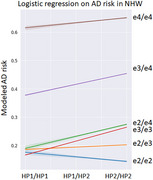# Haptoglobin Structural Variant Modifies APOE Risk Effect in 5155 Non‐Hispanic White and 4133 African Americans

**DOI:** 10.1002/alz70855_106626

**Published:** 2025-12-25

**Authors:** Yuchen Yang, Haimeng Bai, Penelope Benchek, Logan Dumitrescu, Timothy J. Hohman, Kara L Hamilton‐Nelson, Anthony J Griswold, Eden R. Martin, Gerald D. Schellenberg, Richard Mayeux, Lindsay A. Farrer, Margaret Pericak‐Vance, Jonathan L Haines, William S Bush

**Affiliations:** ^1^ Case Western Reserve University, Cleveland, OH, USA; ^2^ Cleveland Institute for Computational Biology, Department of Population and Quantitative Health Sciences, Case Western Reserve University, Cleveland, OH, USA; ^3^ Case Western Reserve Univeristy, Cleveland, OH, USA; ^4^ Vanderbilt Memory & Alzheimer's Center, Vanderbilt University Medical Center, Nashville, TN, USA; ^5^ Department of Neurology, Vanderbilt Memory & Alzheimer's Center, Vanderbilt University Medical Center, Nashville, TN, USA; ^6^ John P. Hussman Institute for Human Genomics, University of Miami Miller School of Medicine, Miami, FL, USA; ^7^ Penn Neurodegeneration Genomics Center, Perelman School of Medicine, University of Pennsylvania, Philadelphia, PA, USA; ^8^ Department of Neurology and the Taub Institute for the Study of Alzheimer's Disease and the Aging Brain, Columbia University Irving Medical Center, New York, NY, USA; ^9^ Boston University Alzheimer's Disease Research Center, Boston University Chobanian & Avedisian School of Medicine, Boston, MA, USA; ^10^ Department of Population & Quantitative Health Sciences, School of Medicine, Case Western Reserve University, Cleveland, OH, USA; ^11^ Department of Population & Quantitative Health Sciences, Case Western Reserve University School of Medicine, Cleveland, OH, USA

## Abstract

**Background:**

A structural variant (SV) of haptoglobin (*HP*) was shown to interact with apolipoprotein E (*APOE*) genotypes to modify Alzheimer's disease (AD) risk effect. The two‐tandem exon repeat of *HP1* to *HP2* affects the structure and antioxidative function of the *HP* protein. *HP* protein has been found to bind *APOE* in the brain tissue of human AD patients, and is able to bind amyloid‐beta in vitro. Our previous discovery was conducted in an array‐genotyped European‐descent cohort, and here we replicate this with whole‐genome sequenced non‐Hispanic White (NHW) and African‐American (AFR) datasets.

**Methods:**

*HP*1/*HP*2 alleles were imputed from 5155 NHW and 4133 AFR samples in the Alzheimer's Disease Sequencing Project using a published panel. We encoded *HP* status as the number of *HP*2 alleles. Each allele of *APOE* was encoded separately as *APOE*1 and *APOE*2, and as a single dosage variable ε2‐ε3‐ε4, assigning *APOE* ε2 baseline risk, with linearly additive risk in ε3 and ε4. Logistic regression was used to model AD associations, *HP*‐by‐*APOE* interactions, and a three‐way term for *HP*‐*APOE*1‐*APOE*2. We accounted for deviations from additivity with stratified analysis for *APOE* carriers of each allele. Age‐of‐onset, sex, and 3 principal components are used as covariates.

**Results:**

The imputed *HP*2 SV frequency was 0.38 in NHW and 0.59 in AFR. Although *HP* did not independently associate with AD, we detected interaction effects between *HP*2 and *APOE* on AD risk. Increase in *HP*2 allele count increased the risk effect of *APOE* ε4 and protective effect of *APOE* ε2 (NHW OR=1.43, *p* = 0.083; AFR OR=1.50, *p* = 0.029). In analysis stratified by one *APOE* allele, we find the largest interaction effect between *HP* and the remaining *APOE* allele (*HP*‐*APOE*‐alt) in *APOE* ε2 carriers (NHW OR=1.31, *p* = 0.121; AFR OR=1.43, *p* = 0.025). Modeled AD risk is lowest in individuals homozygous for both *APOE* ε2 and *HP*2.

**Conclusion:**

We find that a structural variant of *HP* modifies the risk effect of *APOE* alleles. Our findings in an independent dataset corroborate elements of our previous discovery of this interaction effect.